# Psychosocial Underpinnings of Pain and Sleep Disturbance in Safety-Net Primary Care Patients

**DOI:** 10.1155/2020/5932018

**Published:** 2020-04-29

**Authors:** Sarah Griffin, Joseph Tan, Paul B. Perrin, Allison B. Williams, Erin R. Smith, Bruce Rybarczyk

**Affiliations:** ^1^Department of Psychology, Virginia Commonwealth University, Richmond, VA, USA; ^2^Hunter Holmes McGuire Veterans Affairs Medical Center, Richmond, VA, USA

## Abstract

**Objective:**

The aim of this study was to uncover possible psychosocial underpinnings of pain and sleep disturbance in a safety-net primary care sample.

**Methods:**

Patients (*n* = 210) awaiting care in a safety-net primary care clinic waiting room completed measures of cynical hostility, social support, mental health, sleep disturbance, and pain. This study was cross-sectional and observational.

**Results:**

A structural equation model suggested that higher cynical hostility was associated with lower social support, which in turn was associated with poorer mental health, which then corresponded with higher pain and sleep disturbance. All possible indirect (mediational) effects within this model were statistically significant, suggesting a possible route through which cynical hostility may shape pain and sleep, two common presenting problems in primary care.

**Conclusions:**

These findings illustrate the interplay of psychosocial factors with chronic pain and sleep disturbance in a sample of low-income, predominantly African-American patients seeking care at a safety-net primary care clinic. The findings support integrated primary care as a way to target not only behavioral health issues but also the psychosocial factors entangled with physical health.

## 1. Introduction

Chronic pain and sleep disturbance are two of the most common presenting problems in primary care [1]. An estimated 25.3 million Americans experience daily chronic pain [2] and 46.2 million Americans experience sleep disturbance [3]. Chronic pain in primary care is commonly treated with both prescription and over-the-counter medications, including nonsteroidal anti-inflammatory drugs (NSAIDs), paracetamol, cox-inhibitors, and opioids [4]. Sleep disturbance is commonly treated in primary care with sleep hygiene [5] or pharmaceuticals such as benzodiazepines or antidepressants (e.g., trazadone) [6, 7].

There is a growing base of evidence supporting behavioral health interventions in primary care for both pain and sleep disturbance [8, 9]. Further, the field of health psychology outlines several psychosocial factors potentially contributing to these problems. The psychosocial vulnerability model argues that cynical hostility, an attitude characterized by distrust toward others, erodes people's health by weakening social support [10, 11], and research has supported this connection [11, 12]. Another critical route through which cynical hostility and social support may influence health is mental health [13–16]. Social support has been shown to be a protective factor in mental health [17], which in turn predicts both pain [18] and sleep disturbance [19, 20]. Moreover, recent research, for example, work connecting alexithymia—the inability to identify and articulate emotions—to a range of chronic pain conditions shows the importance of psychological factors in problems that are commonly present in medical settings [21].

The present study aimed to elucidate the psychosocial factors underlying pain and sleep disturbance in a primary care sample. We hypothesized that cynical hostility would dampen social support, consistent with the psychosocial vulnerability model, which would in turn correspond with poor mental health (i.e., depression and anxiety), thereby increasing sleep disturbance and pain. The sample for this study consisted of patients seeking primary care services in a safety-net clinic providing care for low-income or homeless persons. Lower socioeconomic status is a predictor for both chronic pain [22] and sleep disturbance [23], as well as poor health and mortality more broadly [24], making the study of this population particularly important in light of health disparities in the United States [25].

## 2. Methods

### 2.1. Participants

The study sample included 210 adults (60% women) recruited from an urban, safety-net primary care clinic (Tables 1 and 2). Racial/ethnic sample composition included 64% Black/African-American, 27% White/European-American, 4% multiracial, 2% Latino/Hispanic, and 2% others. Participants were predominately low-income, with 70% reporting a total personal income of less than $5,000 annually. Participants were included if they were patients of the clinic and excluded if they did not meet health literacy requirements (score ≥10 on the Brief Health Literacy Screening Tool).

### 2.2. Measures

Anxiety was assessed using the Generalized Anxiety Disorder-7, a seven-item measure of symptoms of anxiety (GAD-7) [26]. Items are anchored over a two-week period, using a scale from 0 (Not at all) to 3 (Nearly everyday). Total scores range from 0 to 21, with higher scores suggesting more severe anxiety symptoms. The GAD-7 has shown good internal consistency (*α* = 0.92), test-retest reliability, and convergent validity [26]. The consistency was excellent in the current sample (*α* = 0.93).

Cynical hostility was assessed using five items from the Cook–Medley Hostility Inventory [15]. Participants endorsed agreement with items using a scale from 1 (Strongly disagree) to 5 (Strongly agree). These values were then averaged to generate an index for cynical hostility, ranging from 1 to 6 [27]. The scale showed good internal consistency (*α* = 0.80).

Depression was assessed using the nine-item, Patient Health Questionnaire-9 that includes both somatic and cognitive symptoms (PHQ-9) [28]. Items are anchored over a two-week period, using a scale from 0 (Not at all) to 3 (Nearly everyday). Total scores range from 0–27, with higher scores indicating greater depressive symptomology. The PHQ-9 has been shown to have good internal consistency [28] and is highly correlated with a depression diagnosis in the general population [29]. The reliability in the current sample was good (*α* = 0.88).

Pain was assessed using a single item from the Short Form-12 (SF-12) Health Survey [30]: “During the past 4 weeks, how much did pain interfere with your normal work [including both work outside the home and housework]?.” The response scale for the pain item ranges from 1 (Not at all) to 5 (Extremely), with scores transformed to a 0–100 scale and higher scores representing worse pain.

Sleep was assessed using the four-item, PROMIS Sleep Disturbance-Short Form 4a, which measures perception of aspects of sleep (e.g., quality and ease of falling asleep) [31]. Items are anchored over a seven-day period, using a scale with five response options that vary depending on the item but that maintain the same range in values [1–5]. Total scores range from 4–20, with higher scores indicative of greater sleep disturbance. The measure has demonstrated excellent internal consistency (range: 0.88–0.95) and construct validity in ethnically diverse community samples [32]. The scale demonstrated good internal consistency in the current sample (*α* = 0.87).

Social support was assessed using the 12-item, Interpersonal Support Evaluation List-12 [33], which assesses functional support, including appraisal, belonging, and tangible social support (ISEL-12). Participants endorsed agreement with items using a scale from 0 (Definitely false) to 3 (Definitely true). Total scores range from 0 to 36, with higher scores representing greater social support. The scale demonstrated good internal consistency (*α* = 0.81).

### 2.3. Procedure

Participants were recruited via verbal group announcement from the waiting area of a safety-net primary care clinic from October 2015 to August 2016. As a result, the exact numbers of potential participants approached about recruitment is unknown. The purposes of the survey—to better understand current patient needs and experiences that may affect health—were described as an announcement and patients could express interest in participation. Interested participants provided informed consent and were asked to complete a paper survey while in the waiting area. Participants were compensated $10. This study was approved by Virginia Commonwealth University's Institutional Review Board.

### 2.4. Data Analysis

The means and standard deviations, as well as bivariate correlations were analyzed for all observed variables. To best address any potential biases due to missing data, full information maximum likelihood (FIML) methods were employed. Because these procedures have been found to yield the least biased estimates when all available data are used [34], the entire sample of 210 was used for the purposes of analyses. Missing data were extremely minimal with the following number of participants having complete data on each variable: depression (206), anxiety (208), sleep disturbance (207), hostility (208), pain (208), and social support (209). We conducted structural equation modeling analyses using the package lavaan [35] in R.3.4.4 [36]. We used items as indicators to estimate latent factors for hostility, anxiety, depression, and sleep disturbance. A latent factor for total social support was estimated using the scores for each scale of social support, and a second-order latent factor for mental health was estimated from the latent factors for anxiety and depression. To assess how well the measurement models fit the data, the following indices were used: the comparative fit index (CFI), the Tucker-Lewis Index (TLI), the root mean square error of approximation (RMSEA), and the standardized root mean square residual (SRMR). We did not specify cutoff points to evaluate model fit, as researchers have cautioned against the use of such cutoffs [37, 38].

After establishing adequate fit of each measurement model to the data, we fit our full structural models to test our hypotheses. We assessed model fit of our structural model with the same statistics used to evaluate the measurement model. Indirect effects from hostility to sleep disturbance and pain through mental health and social support were assessed using bias-corrected bootstrapped confidence intervals (with 1000 bootstrap samples) around the standardized indirect effect [39]. We determined a significant indirect effect if the 95% confidence interval of the standardized specific indirect effect did not include 0.

## 3. Results

### 3.1. Descriptives and Bivariate Correlations

Sample characteristics are presented in Table 1 (demographic variables) and Table 2 (psychosocial and health variables). Bivariate correlations are presented in Table 3.

### 3.2. Structural Equation Models

#### 3.2.1. Mediation model

**Figure 1:**
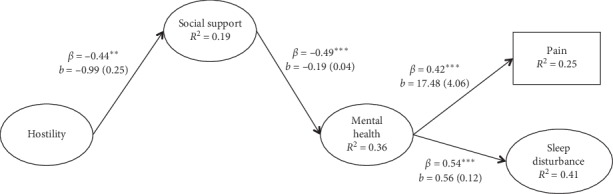
Model testing study hypotheses. *Note*. Unstandardized coefficients are represented by *b* and standardized coefficients are represented by *β*. Standard errors are displayed in parentheses next to the unstandardized coefficients. Nonstatistically significant paths are not shown. ^*∗*^*p* < 0.05. ^*∗∗*^*p* < 0.01. ^*∗∗∗*^*p* < 0.001.

Figure 1 shows a visual representation of the model. Our measurement model showed acceptable fit to the data (χ^2^ (*df* = 342, *N* *=* 210) = 723.75, *p* < 0.001; CFI =  0.89, TLI = 0.87, RMSEA = 0.07 (90% CI for RMSEA = 0.07, 0.08), SRMR = 0.06). Our structural model also demonstrated acceptable fit to the data (χ^2^ (*df* = 366, *N* *=* 210) = 772.99, *p* < 0.001; CFI = 0.88, TLI = 0.87, RMSEA = 0.07 (90% CI for RMSEA = 0.07, 0.08), SRMR = 0.06). Cynical hostility was associated with lower social support (*β* = −0.44, *p* < 0.001). Social support was associated with lower levels of mental health symptoms (*β* = −0.49, *p* < 0.001); cynical hostility was not significantly associated with mental health symptoms when included in the same model (*β* = 0.16, *p*=0.063). Mental health symptoms were associated with higher sleep disturbance (*β* = 0.54, *p* < 0.001) and higher pain ratings (*β* = 0.42, *p* < 0.001).

Cynical hostility was indirectly associated with higher pain ratings through social support and mental health symptoms (standardized indirect effect = 0.09; 95% CI for standardized indirect effect = 0.03, 0.15). Cynical hostility was indirectly associated with higher sleep disturbance through social support and mental health symptoms (standardized indirect effect = 0.12; 95% CI for standardized indirect effect = 0.05, 0.18). Cynical hostility was not significantly associated with sleep disturbance (*β* = 0.06, *p*=0.501), or pain (*β* = 0.09, *p*=0.333), after taking into account the mediating effects of social support and mental health symptoms, indicating full mediation. Indirect effects from cynical hostility to sleep disturbance and pain through just mental health symptoms (i.e. leaving out social support) were not significant. The model explained 41% of the variance in sleep disturbance and 25% of the variance in pain ratings.

## 4. Discussion

The purpose of this study was to uncover potential psychosocial underpinnings of pain and sleep disturbance in a safety-net primary care sample. A structural equation model suggested that higher cynical hostility was associated with lower social support, which in turn was associated with poorer mental health, which then corresponded with higher pain and sleep disturbance. All possible indirect (mediational) effects within this model were statistically significant, suggesting a possible route through which cynical hostility may shape pain and sleep, two common presenting problems in primary care: cynical hostility corrodes social support, which in turn damages mental health, resulting in higher levels of pain and sleep disturbance.

The first finding, that higher cynical hostility was associated with lower social support, is consistent with the psychosocial vulnerability model [10–12]. Previous longitudinal work shows that hostility predicts subsequent deterioration of personal relationships [40, 41], indicating that hostility weakens social support over time. The finding that social support was inversely associated with mental health problems, in particular depressive symptoms, is well-documented in the literature [17, 42, 43]. Similarly, there is strong evidence for the association between mental health problems and both sleep disturbance and chronic pain. Longitudinal research shows reciprocal effects [44]; although the majority of studies have focused on insomnia symptoms as a predictor of depression [19] and anxiety, there is evidence that depression predicts subsequent insomnia. A recent meta-analysis showed bidirectional prediction between sleep disturbance and depression in older adults [45]. Moreover, although the bulk of studies examining the longitudinal relationship between mental health and pain has focused on mental health, in particular depression, as a risk factor for subsequent chronic pain, there is also evidence that chronic pain predicts subsequent depression [46].

This study found positive associations between cynical hostility and anxiety/depression in the correlation analysis. However, in the full model, there was a significant indirect effect—whereby social support fully mediated the relationship between cynical hostility and mental health. The detection of an association between hostility and mental health is consistent with the existing research [13, 14, 47]. However, the finding that social support fully mediates the link between cynical hostility and mental health differs from past findings: two longitudinal studies found that hostility predicted subsequent depressive symptoms when accounting for social support [13, 14]. It is possible that this difference in findings is due to the sample used by this study, which was predominantly low-income: Nabi and colleagues [14] found that adjusting for socioeconomic status attenuated the relationship between hostility and depression. Social support may be particularly vital in mitigating the effects of cynical hostility on mental health in low-income patients. The detection of an indirect effect of hostility on pain and sleep disturbance via social support and mental health is consistent with previous research [48–51].

This study builds upon the previous literature through investigating a holistic model of hostility, social support, mental health, pain, and sleep disturbance. These factors do not exist in a vacuum, but rather may interplay to manifest as complaints of chronic pain and sleep disturbance in primary care. Research examining models of symptomatology—rather than individual risk factors—is necessary to inform treatment of real-world patients who present with a myriad of issues. Further, these findings underscore the importance of integrated care. Integrated care allows for intervention at multiple levels of the current model, with potential cascading benefits on other domains. For example, the incorporation of psychologists in primary care not only provides empirically based treatments for both chronic pain and sleep disturbance, it also provides assessment and brief-intervention—or referral—for psychosocial factors connected with these issues, to include cynical world-view, weak social support, and symptoms of depression and anxiety.

This sample consisted of primarily low-income and Black Americans, a population which has been shown to have higher levels of cynical hostility [52]. Cynical hostility may represent an adaptive coping response in this population. Ecosystem distrust—mistrust of one's surroundings, to include people and institutions—may be a natural byproduct of exploitation, victimization, and disappointment over the lifespan that protects against further harm to self, particularly if homeless [53]. However, the health risks remain [54]. Furthermore, this social posture may shape interactions with clinicians and the broader health system. A potential key aim for integrated care in safety-net clinics is to establish trust where possible with clinical staff, thus mitigating further health consequences of ecosystem distrust in vulnerable populations. The theory of ecosystem distrust also provides an alternative explanation for why social support fully mediates the connection between cynical hostility and mental health in this sample: cynical hostility may be an adaptive world-view in the face of poverty and discrimination, but social support is still vital to mental health. As such, another potentially useful treatment target in integrated care settings is working with patients to selectively build trusting relationships despite worldviews incorporating higher levels of distrust.

This study has several limitations. First, it is cross-sectional and therefore cannot speak to the longitudinal relationships among cynical hostility, social support, mental health, pain, and sleep disturbance. As such, it is not possible to speak conclusively to the direction of effects. It is likely that several of the associations in the model are reciprocal, whereby both factors are contributing to the other. Second, this study is observational and therefore cannot speak to causality. Third, this study did not examine stress, which is likely another important component of this model. Further research is necessary to replicate these findings and speak to direction of effects. Specifically, longitudinal research examining the interplay of hostility, social support, mental health issues, pain, sleep disturbance, and stress would allow for the development of a comprehensive model which speaks to the temporal relationships between these variables, thereby identifying early treatment targets.

This study illustrates the interplay of psychosocial factors with chronic pain and sleep disturbance in a sample of low-income, predominantly African-American patients seeking care at a safety-net primary care clinic. This study suggests that cynical hostility weakens social support, which in turn damages mental health to exacerbate chronic pain and sleep disturbance. These findings support multifaceted treatment targeting not only chronic pain and sleep disturbance, but the psychosocial factors underlying these problems. Integrated primary care is a way to target not only behavioral health issues but also the psychosocial factors entangled with physical health. The accumulation of evidence documenting comorbidity across conditions—across what is deemed physical, social, psychological—steadily grows. The present paper echoes this literature to call for holistic conceptualization and treatment of primary care patients.

## Figures and Tables

**Table 1:** Sample characteristics: demographics

Variable	
*Categorical*	*n* (%)
Sex	
Male	84 (40%)
Female	126 (60%)
Education	
Elementary school	1 (0.5)
Middle school/Junior high	18 (8.6)
High school	111 (53.1)
Some community college (no degree)	52 (24.9)
2-year/technical degree	7 (3.3)
4-year college degree	17 (8.1)
Master's degree	3 (1.4)
Race	
Asian/Asian-American/Pacific Islander	1 (0.5)
Black/African-American (non-Latino)	134 (63.8)
Latino/Hispanic	4 (1.9)
American-Indian/Native-American	3 (1.4)
White/European-American (non-Latino)	57 (27.1)
Multiracial/multiethnic	9 (4.3)
Others	2 (1.0)
Income	
$0–$4,999	146 (69.9)
$5,000–$9,999	29 (13.9)
$10,000–$14,999	13 (6.2)
$15,000–$19,999	9 (4.3)
$20,000–$24,999	4 (1.9)
$25,000–$29,999	4 (1.9)
$30,000+	4 (1.9)
*Continuous*	*Mean (standard deviation)*
Age	44.69 (11.57)
Range	21 to 67

**Table 2:** Sample characteristics: biopsychosocial health factors.

Variable	
*Continuous*	*Mean (standard deviation)*
Depression	11.22 (6.85)
Scale range	0 to 27
Anxiety	9.75 (6.69)
Scale range	0 to 21
Sleep disturbance	13.48 (4.22)
Scale range	4 to 20
Hostility	3.72 (1.28)
Scale range	1 to 6
Pain	45.79 (35.83)
Scale range	0 to 100
Social support	10.22 (2.48)
Range	0 to 36

**Table 3:** Correlations with confidence intervals.

Variable	1	2	3	4	5	6
1. Gender						
2. Depression	0.18^*∗∗*^					
	[0.05, 0.31]					
3. Anxiety	0.17^*∗*^	0.80^*∗∗*^				
	[0.04, 0.30]	[0.75, 0.85]				
4. Sleep disturbance	0.11	0.58^*∗∗*^	0.55^*∗∗*^			
	[−0.03, 0.24]	[0.48, 0.66]	[0.44, 0.63]			
5. Hostility	0.09	0.34^*∗∗*^	0.34^*∗∗*^	0.29^*∗∗*^		
	[−0.04, 0.23]	[0.22, 0.46]	[0.21, 0.45]	[0.16, 0.41]		
6. Pain	0.19^*∗∗*^	0.43^*∗∗*^	0.45^*∗∗*^	0.41^*∗∗*^	0.26^*∗∗*^	
	[0.06, 0.32]	[0.31, 0.54]	[0.33, 0.55]	[0.28, 0.51]	[0.13, 0.38]	
7. Social support	−0.01	−0.50^*∗∗*^	−0.43^*∗∗*^	−0.40^*∗∗*^	−0.35^*∗∗*^	−0.30^*∗∗*^
	[−0.15, 0.12]	[−0.60, −0.39]	[−0.54, −0.31]	[−0.51, −0.28]	[−0.46, −0.22]	[−0.42, −0.17]

*Note*. Values in square brackets indicate the 95% confidence interval for each correlation. The confidence interval is a plausible range of population correlations that could have caused the sample correlation. ^*∗*^*p* < 0.05. ^*∗∗*^*p* < 0.01.

## Data Availability

Data are available for review by contacting the corresponding author.
